# A Novel Transwell Blood Brain Barrier Model Using Primary Human Cells

**DOI:** 10.3389/fncel.2019.00230

**Published:** 2019-06-06

**Authors:** Nicole L. Stone, Timothy J. England, Saoirse E. O’Sullivan

**Affiliations:** Division of Medical Sciences and Graduate Entry Medicine, School of Medicine, University of Nottingham, Nottingham, United Kingdom

**Keywords:** blood-brain barrier, transwell, *in vitro*, BBB model, BBB permeability, primary human cells, stroke

## Abstract

Structural alterations and breakdown of the blood brain barrier (BBB) is often a primary or secondary consequence of disease, resulting in brain oedema and the transport of unwanted substances into the brain. It is critical that effective *in vitro* models are developed to model the *in vivo* environment to aid in clinically relevant research, especially regarding drug screening and permeability studies. Our novel model uses only primary human cells and includes four of the key cells of the BBB: astrocytes, pericytes, brain microvascular endothelial cells (HBMEC) and neurons. We show that using a larger membrane pore size (3.0 μM) there is an improved connection between the endothelial cells, astrocytes and pericytes. Compared to a two and three cell model, we show that when neurons are added to HBMECs, astrocytes and pericytes, BBB integrity was more sensitive to oxygen-glucose deprivation evidenced by increased permeability and markers of cell damage. Our data also show that a four cell model responds faster to the barrier tightening effects of glucocorticoid dexamethasone, when compared to a two cell and three cell model. These data highlight the important role that neurons play in response to ischaemia, particularly how they contribute to BBB maintenance and breakdown. We consider that this model is more representative of the interactions at the neurovascular unit than other transwell models and is a useful method to study BBB physiology.

## Introduction

The blood brain barrier (BBB) is a unique interface that separates the peripheral blood supply and neuronal tissue. Structurally, the BBB is comprised of specialized brain microvascular endothelial cells (HBMECs), perivascular cells (pericytes) and astrocytes (Abbott et al., 2006, 2010). Neurons and microglia also contribute to the maintenance of the BBB and form what is known as the neurovascular unit (NVU) (Abbott et al., 2006). Pericytes contribute 22–32% of the cerebral vasculature and together with vascular smooth muscle cells and endothelial cells they maintain vascular function (Martini and Bartholomew, 2017). In the CNS, pericytes are present at a higher ratio to HBMECs in the brain compared to the periphery and recent studies have shown the extensive role of pericytes in BBB development and maintenance (Kacem et al., 1998; Armulik et al., 2011; Sweeney et al., 2016). As well as offering mechanical support, they also regulate vessel contractility, endothelial proliferation, blood flow and angiogenesis (Bergers and Song, 2005; Dore-Duffy, 2008). Pericytes have also been shown to secrete angiogenic factors such as vascular endothelial growth factor (VEGF), that support endothelial cell survival and proliferation (Darland et al., 2003). In pathologies such as ischaemic stroke, large gaps can develop between adjacent pericytes, increasing barrier permeability and vessel leakage. These alterations in pericyte morphology, coupled with an upregulation in the expression of adhesion molecules and leukocyte integrin ligands, contribute to the extravasation of peripheral leukocytes into the brain following ischaemic insult (Pieper et al., 2013). Thus, pericytes play a large role in cerebral vascular function under normal physiological conditions as well as vascular dysfunction in hypoxia.

Several studies have also highlighted the roles of neurons and glia in BBB development and maintenance. Neural progenitor cells present in the ventricular neuroepithelium have been shown to aid endothelial cell recruitment during early BBB development, which is largely governed by the Wnt signaling pathway (Risau et al., 1986). Specifically, Wnt signaling in early CNS development is responsible for vascular stabilization and angiogenesis (Liebner et al., 2008). Further to this, early neuronal signaling has been shown to be essential for the maturation of the BBB, specifically, tight junction (TJ) organization. A study carried out using rat microvascular endothelial cells and neuronal progenitor cells, showed that in the presence of neural progenitor cells, endothelial cells established regular TJ formation including: claudin 5, zonula occludens (ZO-1) and occludin (Weidenfeller et al., 2007). After maturation, maintenance of the BBB and preservation of brain homeostasis is largely dependent on adequate perfusion to neuronal tissue and neuronal signaling to cerebral vessels, a process known as hyperaemia (Attwell et al., 2010). Studies have shown that neuronal-astrocyte crosstalk is important for appropriate vessel contractility and blood flow, depending on metabolic demand (Zonta et al., 2003; Attwell et al., 2010; Macvicar and Newman, 2015). In cerebral ischaemia, astrocytes sense elevations in Ca^2+^ ions and increases in extracellular glutamate released by neurons and respond accordingly, secreting a range of vasoactive substances to help mitigate the effects of the blood vessel occlusion (Macvicar and Newman, 2015). Altogether, interactions between both neural and vascular cells within the NVU is considered to be paramount in BBB functionality because together they induce and strengthen barrier properties; helping to maintain its key features including low paracellular permeability and functional tightness (Abbott et al., 2010). Breakdown of the BBB in conditions such as ischaemic stroke can lead to severe consequences to brain homeostasis, therefore, modeling these interactions is necessary to understand the complex signaling networks between these cell types and how they are influenced in disease states.

**FIGURE 1:**
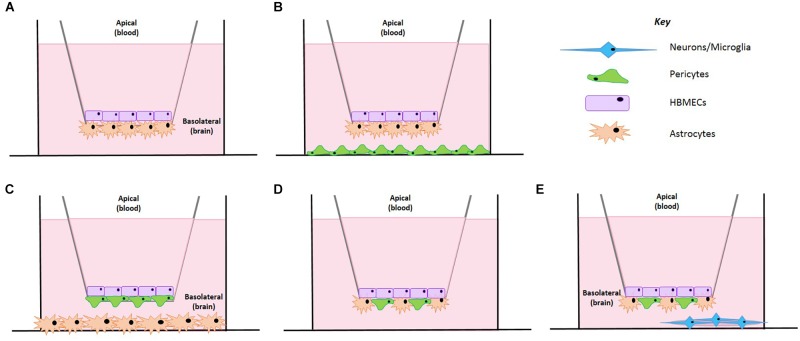
Schematic representation of the BBB model development. **(A)** A co-culture cell model containing HBMECs and astrocytes. **(B)** HBMECs seeded on the apical side, astrocytes seeded on the underside of the insert and pericytes seeded on the plate bottom. **(C)** HBMECs seeded on the apical side of the insert, pericytes seeded on the underside of the insert and astrocytes seeded on the plate bottom. **(D)** HBMECs seeded on the apical side of the insert with mixed culture of astrocytes and pericytes on the underside of the insert. **(E)** HBMECs seeded on the apical side of the insert with mixed culture of astrocytes and pericytes on the underside of the insert and neurons seeded on a poly-L-lysine coated coverslip on the plate bottom.

To date, a number of BBB models have been developed ranging from HBMEC monolayers to more sophisticated spheroid and chip style models, see Table 1. After the successful isolation of brain endothelial cells, the first, most simplistic BBB models were developed utilizing HBMECs as a single monolayer in the abluminal side of transwell inserts, see Table 1 (Borges et al., 1994; Hartz et al., 2010). Later addition of other BBB cell types (namely astrocytes and pericytes), led to the development of co-culture transwell systems which exhibited greater barrier strength, exhibited by higher transepithelial resistance (TEER) and lower permeability than single HBMEC models, see Figure 1. More recent transwell systems typically use three cell types originating from either bovine, porcine or rodent origin, see Table 1 (Gaillard et al., 2000; Nakagawa et al., 2009; Thomsen et al., 2015).

**Table 1:** Different models of the blood brain barrier; their features, advantages, and disadvantages.

Model type	Typical components	Advantages	Limitations	Representative of BBB phenotype	References
Single-cell transwell systems (non-co-culture)	A monolayer of HBMECs cultured in the apical compartment of the transwell insert.	Very easy to set up. Minimal cost. Low labor intensity. Useful if wanting to study endothelial cells alone.	TEER is typically low.	Cobblestone appearance of HBMECs, barrier formation. Little information on the impact of additional cell types.	Borges et al., 1994; Hartz et al., 2010
Co-culture /multicellular transwell systems	HBMECs cultured on the apical side of the transwell insert and astrocytes and/or pericytes cultured on the underside of the transwell insert.	Time and cost effective. Higher TEER. Greater barrier stability.	Some models are not fully in contact.	Closer representation of the BBB with the addition of important cell types. Able to study interactions between cell types and how they influence BBB phenotype.	Hind, 2014; Wang et al., 2015; Appelt-Menzel et al., 2017
Spheroid	3D organization of cells typically using matrigel. Typically consists of HBMECs and astrocytes and/or pericytes with some models containing neuronal cell types.	3D Cell model. No scaffold. Reduced de-differentiation.	Cannot measure permeability with this model. Expensive and greater skill required.	Microvessels wrap around endothelial cells and provide structural support. Helps to induce tight junction proteins. Closely represents the *in vivo* set up with cells in direct contact with each other. Applications include: cancer drug and neurotoxicity screening.	Cho et al., 2017; Nzou et al., 2018
Microfluidic systems/3D chip-style models	3D organization of cells with the added benefit of a “flow” system to mimic cerebral blood flow. Typically consists of HBMECs and astrocytes and/or pericytes with some models containing neuronal cell types.	Advantage of mimicking sheer stress which is essential for HBMECs optimum phenotype.	Difficult to set up and maintain adequate flow unless linked to a computer system.	Useful to assess the impact of blood flow on cell development and optimum phenotype. Also useful in studying cell migration and metastatic progression.	Yeon et al., 2012; Wang et al., 2017

*HBMECs = human brain microvascular endothelial cells, TGFβ = transforming growth factor beta, TEER = transepithelial resistance, BBB = blood brain barrier.*

Whilst modeling using non-human cells is cheaper and easier to obtain, they are not comparable to human cells, with many studies showing key differences in morphology and function, particularly their sensitivity to glutamate and expression of efflux transporter proteins (Oberheim et al., 2009; Warren et al., 2009; Zhang et al., 2016). More complex BBB models are also available, such as spheroid or microfluidic models and offer a closer representation of the *in vivo* environment. However, these set ups are difficult and expensive to assemble (Ruck et al., 2015). Therefore, there is a need to develop a multicellular transwell model that incorporates multiple NVU cell types to study their interactions, particularly the role of neurons and their influence on barrier strength in both physiological and disease states. Transwell systems still offer a distinct advantage in that they are relatively easy to setup and control, as well as offering a range of endpoints to study. Measuring TEER in these types of models is commonplace as it provides a reliable, non-invasive quantitative measure of barrier integrity, enabling repeated measurements to be taken over the desired time period with minimal damage to cells (Srinivasan et al., 2015). Further to this, transwell models enable access to both the apical and basolateral (basal) compartments for drug application and medium sampling as well as being able to visualize cells over the course of the experiment.

Our aim was therefore to create a novel four cell human BBB model to study changes in permeability post oxygen-glucose deprivation (OGD) and for use in *in vitro* pharmacology. We initially focused on model development, refining a protocol first outlined by Hind (2014) by optimizing the inserts themselves, insert coating, cell seeding densities and cell culture timelines. Finally, we incorporated a method of seeding neurons on plastic coverslips which were placed on the bottom of 12 well cell culture plates. Thus, our model maintains the ease of the transwell setup but utilizes four primary human cells, making it a closer representation of the human *in vivo* environment.

## Materials and Methods

Primary cells (astrocytes, pericytes, HBMECs, and neurons) and specialized cell culture medium (astrocyte medium, pericyte medium, endothelial cell medium, and neuronal medium) were obtained from ScienCell, United States supplied by Caltag Medsystems, United Kingdom. Poly-L-lysine and porcine fibronectin were also obtained from ScienCell, United States supplied by Caltag Medsystems, United Kingdom. Collagen coated inserts, 3.0 μm, 12 mm were obtained from Corning, United Kingdom. Plastic coverslips (Thermanox^^®^^ 13 mm diameter), Accutase dissociation reagent and glucose free RPMI medium were obtained from Thermo Fisher Scientific, United Kingdom.

Cells were maintained in a humidified incubator (37°C, 5% CO_2_). Astrocytes and pericytes were cultured and used between passages 4 and 6. Human brain microvascular endothelial cells (HBMECs) were used between passages 3 and 5 and neurons were used at passage 1. During subculture, flasks containing HBMECs were coated with 2 μg⋅cm^2^ of fibronectin before reviving or splitting cells as per manufacturers recommendations. Cells were passaged at 80–90% confluency. Inserts contained 1.2 mL of medium in the basolateral compartment and 800 μL in the apical compartment.

STX-3 probes and Ohms meter were obtained from World Precision Instruments, United Kingdom. Dexamethasone was obtained from Sigma, United Kingdom and dissolved in DMSO at a stock concentration of 10 mM and subsequently diluted in cell culture medium. GasPak^TM^ EZ anaerobe container systems were obtained from BD, United Kingdom.

### Model Validation

Our model was based on an initial co-culture set up established by Hind (2014) and previous models by Allen and Bayraktutan (2009). Our model was modified and developed in a number of preliminary experiments including comparison of insert pore sizes, insert coating, cell organization and addition of multiple cell types.

### Pore Size, Insert Size and Coating

**FIGURE 2:**
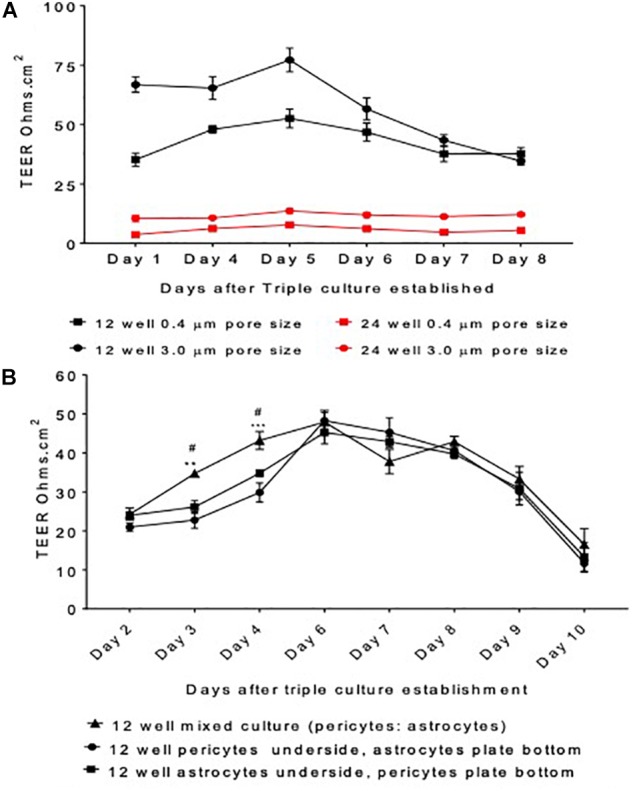
Model protocol development **(A)** measured transepithelial resistance (TEER) as a marker of barrier tightness comparing a 12 well plate transwell set up vs. a 24 well plate transwell set up and insert pore size 3.0 μm vs. 0.4 μm. HBMECs seeded on the apical side of the insert, astrocytes underneath and pericytes on the plate bottom. **(B)** The organization of cells was optimized by comparing the TEER generated by a mixed culture of astrocytes and pericytes, pericytes or astrocytes alone on the underside of transwell inserts and astrocytes or pericytes on the cell culture plate bottom. HBMECs were seeded on the apical side of the insert. Data given as mean ± SEM, *n* = 4–6 from two experimental repeats. Statistical analysis conducted using 2-way ANOVA with Turkey’s multiple comparisons test, ^∗∗^*P* < 0.01 and ^∗∗∗^*P* < 0.001 mixed culture astrocytes and pericytes vs. pericytes underside the insert and astrocytes on plate bottom. ^#^*P* < 0.05 mixed culture astrocytes and pericytes vs. astrocytes underside the insert and pericytes on plate bottom.

Initially, pore sizes of Corning, United Kingdom inserts were compared (0.4 μm vs. 3.0 μm) as well as cell culture plates (12 well vs. 24 well). This was to determine the best initial setup that provided the highest and most stable barrier resistance, as well as giving the best cell contact. During protocol development, we found addition of pericytes in the smaller 24 well plates yielded poor results and insufficient TEER, suggesting inadequate barrier formation. Possibly as a result of inadequate cellular growth in such a small surface area and environment. Therefore, 24 well plates were switched back to 12 well plates, which resulted in substantially higher TEER readings. Following work carried out by Niego and Medcalf (2013), we also found that inserts with a 3.0 μm pore size had higher TEER values than 0.4 μm inserts, suggesting that increased contact between the cells in the apical and basolateral sides of the insert resulted in greater barrier strength, see Figure 2A.

### Addition of Multiple Cell Types and Cell Positioning

Despite these improvements on the co-culture model, the need for additional cell types was critical to create a closer representation of the *in vivo* BBB. We established three different set ups as shown in Figure 1. In one, astrocytes were seeded on the basolateral side of the inserts and pericytes on the bottom of the culture dish (Figure 1B), in another pericytes were seeded on the basolateral of the inserts whilst astrocytes were seeded on the bottom of the culture dish (Figure 1C) and finally the last set up involved a mixed culture of astrocytes and pericytes seeded on the basolateral side of the insert (Figure 1D). In all models tested, HBMECs were seeded in the apical side of the transwell insert.

The final set up offered a closer replication of the organization held at the *in vivo* BBB, as cells would be in direct contact allowing them to exchange vital growth factors required for cellular growth and development. We found that mixed culture of pericytes and astrocytes exhibited significantly higher TEER values when compared to the set-up with pericytes seeded on the plate bottom and astrocytes underneath the insert or astrocytes on the plate bottom and pericytes underneath the insert on days 3 and 4, *P* < 0.05 and *P* < 0.01, respectively (Figure 2B). Furthermore, this set up was also considered the most stable, as shown by steadier TEER readings and was altogether more physiologically relevant. This set up was therefore taken forward in subsequent four cell protocol development.

To test the viability of adding neurons to the model, we originally seeded neurons on the bottom of the 12 well plate in which the inserts were hung. This, however, was not feasible as the TEER probes touched the bottom of the plate causing unwanted damage to the cells. Therefore, we decided to utilize coverslips that could be positioned on the plate bottom, but not take up the entirety of the well, allowing the probe to sit where the cells were not present. After testing both poly-L-lysine coated glass and plastic coverslips, we found that plastic coverslips coated were the most effective in neuronal adhesion and this method was used in the final model.

### Four Cell Method Overview

After optimization, our four cell BBB model consisted of four major NVU cell types arranged in a transwell permeability set-up (see Figure 1E). The assembly of this involves seeding different cell types at different times on the apical and basolateral sides of the transwell insert. During this time, neurons are seeded on plastic coverslips placed on the bottom of a separate 12 well plate to develop neurite before putting both parts of the model together on the final day of model establishment. Cell culture medium in both compartments was replaced every other day and the final set up was left to equilibrate for 2 days before commencing experiments. Greater than 85% of inserts are feasible for use in experiments and the model remained viable for up to 5 days.

#### Insert Coating and Astrocyte Seeding

**FIGURE 3:**
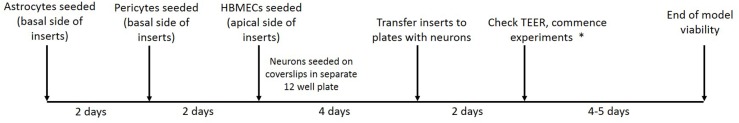
Timeline showing stages of model establishment. On day 1, inserts were coated and astrocyte seeded on the basolateral side of transwell inserts and on day 3 pericytes were seeded on the basolateral side of inserts to form a mixed culture. On day 6 HBMECs were seeded on the apical side of inserts and neurons were seeded on coated plastic coverslips in a separate 12 well plate. On day 10/11, inserts are carefully lifted out of their current plate and placed into the second 12 well plate containing the neurons seeded on coverslips. After 2 days, TEER measurements are taken to ensure adequate barrier formation. ^∗^In our lab OGD experiments were commenced at this point and were viable for 4–5 days.

**FIGURE 4:**
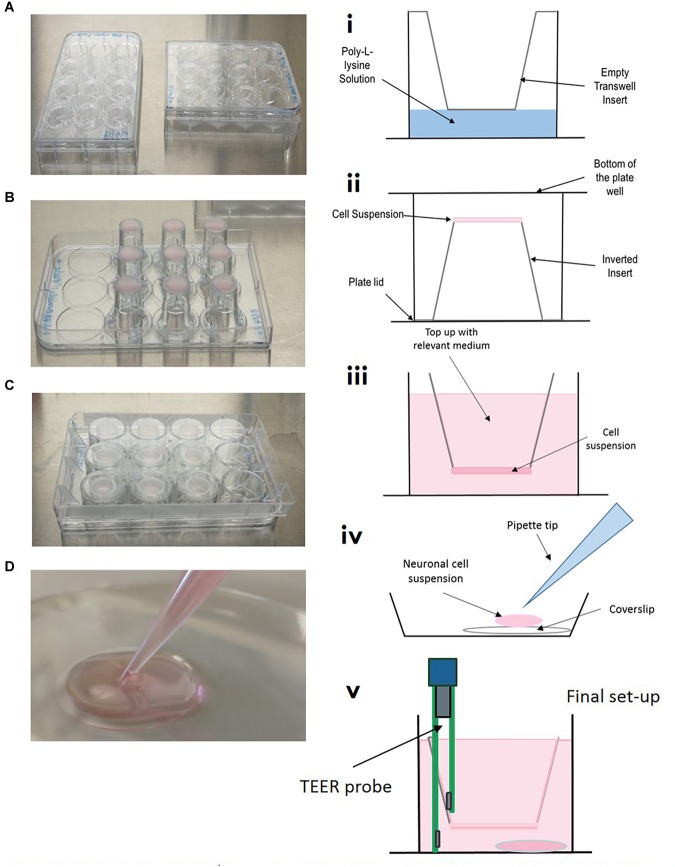
Model setup **(A–D)** and **(i–v)**. **(A/i)** Inserts are placed into 12 well plate, coated with poly-L-lysine and washed, ensuring all of the liquid is removed. **(B/ii)** Inserts are carefully flipped inside the plate and the plate removed. 100 μL of relevant cell suspension is carefully placed on the underside of the insert. **(C/ii)** The bottom of the cell culture plate acts as a “lid” and is replaced as quickly as possible, plates are then returned to the incubator for the cells to adhere for 3–4 h. **(iv)** In a separate 12 well plate, coverslips are placed in the bottom of the culture dish, coated with poly-L-lysine and seeded with neuronal cell suspension. **(v)** Once all cells have been seeded on transwells, inserts are carefully transferred to plates containing neurons on coverslips.

On day one, the basolateral side of transwell inserts were coated with poly-L-Lysine and astrocytes were seeded on the basolateral side of the inserts, see Figure 3. Briefly, 3.0 μm, 12 mm collagen coated inserts (Corning, United Kingdom) were carefully removed from outer packaging and placed into 12 well cell culture plates using sterile forceps. A solution of poly-L-Lysine (2 μg/cm^2^) was prepared in sterile water, homogenously mixed and carefully pipetted using a Pasteur pipette to just cover the basolateral of the insert, see Figure 4A,i. Plates containing inserts were then returned to the incubator, 37°C, 5% CO_2_ for 1 h as per supplier recommendations. After 1 h, plates were removed from the incubator and washed twice with sterile water to remove any residual poly-L-lysine. All remaining liquid was removed by careful aspiration. Transwell inserts were then flipped inside the plate and the lid removed (Figure 4B). On the newly coated inserts, 100 μL of astrocyte cell suspension in astrocyte medium (3.13 × 10^5^ cells) was pipetted quickly onto the basolateral side of the transwell and the lid carefully replaced (see Figure 4C,ii). Plates were returned to the cell culture incubator for 2–3 h for the cells to adhere. After this time, transwell inserts were reverted and any excess medium was removed by aspiration. Medium was topped up in the apical and basolateral compartments, see Figure 4iii. Again, plates were returned to the incubator.

#### Pericyte Seeding

On day 2, plates were removed from the incubator and the astrocyte medium was removed with care so as to not disturb the layer of cells on the basolateral side of the insert. Inserts were then inverted again and 100 μL of 6.25 × 10^4^ pericyte cell suspension was added to the astrocyte cell layer on the basolateral side of the transwell inserts, giving an approximate ratio of 5:1 astrocytes to pericytes (Pardridge, 1999). Plate lids were quickly replaced and returned to the incubator for 2–3 h. After this time, transwell inserts were reverted and any excess medium was removed by aspiration and a mixture of astrocyte and pericyte medium (1:1) was added to the apical and basolateral compartments.

#### HBMEC Seeding

Once astrocytes and pericytes reached 90% confluency (approximately day 4 from model initiation, see Figure 3), the astrocyte:pericyte (1:1) medium in the apical compartment was removed and 100 μL of HBMEC cell suspension (7.5 × 10^4^) in HBMEC medium was added to the apical compartment of transwell inserts and cells were left to adhere for a minimum of 5 h, then medium was topped up to 700 μL with endothelial cell medium and plates returned to the incubator.

#### Neuronal Seeding

On the same day as HBMEC seeding, plastic coverslips (13 mm diameter) were coated with poly-L-lysine and placed in the cell culture incubator for a minimum of 1 h, as per supplier recommendations. Plates containing coverslips were carefully removed from the incubator and coverslips were washed twice with sterile water and left to air dry in the cell culture hood. Following this, cryopreserved neurons were revived into 3 mL of neuronal medium (to give a total cell suspension of 4 mL) and 100 μL of cell suspension was added to each coverslip (thus seeded at a density of approximately 2.5 × 10^4^ cells per cm^2^ within the optimum range according to the manufacturer’s recommendations) (Figure 4D,iv). Medium was topped up after 2 h and half of the medium replaced every 2–3 days. After light microscope observation, neurons began showing extensive neurite growth at approximately day 5. At this point HBMECs will have almost formed a confluent monolayer above the astrocytes and pericytes. Transwells were then carefully lifted out of their current 12 well plate using sterile forceps and placed into the 12 well plate containing the neuronal coverslips. Fresh HBMEC medium was applied to the apical compartment and a mix of pericyte, astrocyte and neuronal medium (1:1:2, respectively) was added to the basolateral compartment. This was to maintain a low concentration of fetal bovine serum optimum for neuronal maintenance, whilst also preserving growth of astrocytes and pericytes. As all cells were confluent and the barrier was adequately formed, conditions were able to be maintained in the different compartments.

### Oxygen-Glucose Deprivation (OGD) Protocol

An oxygen-glucose deprivation (OGD) protocol was used to increase barrier permeability, simulating the effects of ischaemic stroke *in vitro* (Hind et al., 2015, 2016). Normal cell culture medium was removed from transwell inserts and replaced with glucose free RPMI medium (Thermo Fisher Scientific, United Kingdom) and placed in a 0% O_2_ environment (GasPak^TM^ anaerobe pouch Beckton Dickinson, Oxford, United Kingdom) for 20 min to ensure anaerobic conditions for a further 4 h. There was no initial pre-conditioning period. Reperfusion was initiated by removing plates from the anaerobe pouch and returning cells to their normal medium (HBMEC medium in the apical compartment and in the basolateral compartment a mix of pericyte, astrocyte and neuronal medium, 1:1:2, respectively). TEER was measured at baseline (0 h), immediately post OGD (4 h), 24, 48, and 72 h.

#### Evaluation of Barrier Integrity

Transepithelial resistance (TEER) was measured prior to commencing OGD experiments to ensure model barrier integrity; inserts should exhibit a TEER value of ≥45 Ω/cm^2^ (Figure 4v). Light microscope observation was also carried out to ensure cell confluency and successful neurite formation. To ensure consistency, TEER measures should always be read at least 24 h after a medium change. Briefly, STX3 electrodes were sterilized by placing the tips of the probe in 70% ethanol, and then equilibrated for 15 min in endothelial cell culture medium at room temperature. The STX probe was then connected to an EVOM^2^ meter (Both World Precision Instruments, United Kingdom) and inserted into the transwell insert. The electrode has two parts that are uneven in length, the longer part of the electrode was placed so it gently touched the bottom of the cell culture plate, whilst the shorter electrode rested slightly above the insert dish, not quite making contact the HBMEC cell layer. Care should be taken to avoid disrupting the neurons on the bottom of the cell plate, see technical comments and limitations. As TEER values are very susceptible to change, it is important to keep the electrode upright and avoid tilting as this can cause fluctuation in the TEER values. A background reading for an insert with just cell culture medium was taken and subtracted from each reading (readings were repeated twice to ensure reproducibility), this was then multiplied by 1.12 to address the cell culture insert area (cm^2^) (Hind, 2014).

### Dexamethasone Protocol

Dexamethasone is a synthetic glucocorticoid and several groups have shown that is able to artificially improve barrier strength (Shi and Zheng, 2005; Pyrgos et al., 2010; Hind, 2014). Therefore, we used dexamethasone as a positive control to investigate any potential difference in the response of the three versus four cell model to a drug application. Baseline TEER readings were recorded and medium replaced, then dexamethasone was added to the apical compartment of the transwell insert, giving a final concentration of 1 μM. TEER was measured at 2, 4, and 24 h.

### Data Analysis

Data analysis was carried out using GraphPad prism software (La Jolla, CA, United States). Data are presented as mean ± SEM and analyzed using two-way ANOVA, followed by Sidak’s or Turkey’s multiple comparisons test. ^∗^*P* < 0.05 was considered significant.

### Technical Comments and Limitations

A critical step for setting up the four cellular model is timing and the revival and seeding of human neurons. Addition of the cells at incorrect timings will result in the model not working as effectively and TEER values will be lower than anticipated. We have therefore outlined a timeline for setting the model up (Figure 2), steps 4 and 5 can vary depending on the time taken for barrier formation to take place and for neurons to form neurite. Improper technique when seeding neurons on the coverslips will result in a lack of uniformity and inadequate neurite formation. Ensure coverslips are adequately air dried and neuronal cell suspension is carefully but adequately mixed during the revival and seeding process. Avoid removing neurons from the incubator for long periods.

When taking TEER values, ensure that the larger part of the STX probe does not touch the neurons cultured on the coverslip. This is especially important if multiple readings are being made (recommended). Utilization of neurons after primary experiments have been completed is also possible. Staining can be done on the coverslips using a variety of techniques including propidium iodide (PI) and DAPI staining, neurons can be lysed and intracellular assays can be performed.

## Results

### Protocol Development

During BBB model development various set ups were compared including; insert pore size, plate size, and cell organization. Figure 2 highlights stages in protocol development and their respective TEER values, prior to the addition of neurons into the model. Figure 2A shows that a larger pore size (3.0 μm) exhibited greater barrier integrity (as shown by greater TEER readings) than the smaller pore size (0.4 μm). Furthermore, the 12 well inserts displayed considerably greater TEER readings than the 24 well inserts. Continuing model development using 12 3.0 μm inserts, Figure 2B compares three different cell culture set-ups days after model establishment. On days three and four, the inserts containing a mixed culture of astrocytes and pericytes displayed significantly higher TEER readings than set-ups containing astrocytes or pericytes seeded on the underside of the inserts or the cell culture plate bottom, *P* < 0.05 and *P* < 0.01, respectively.

### OGD Model Simulation

**FIGURE 5:**
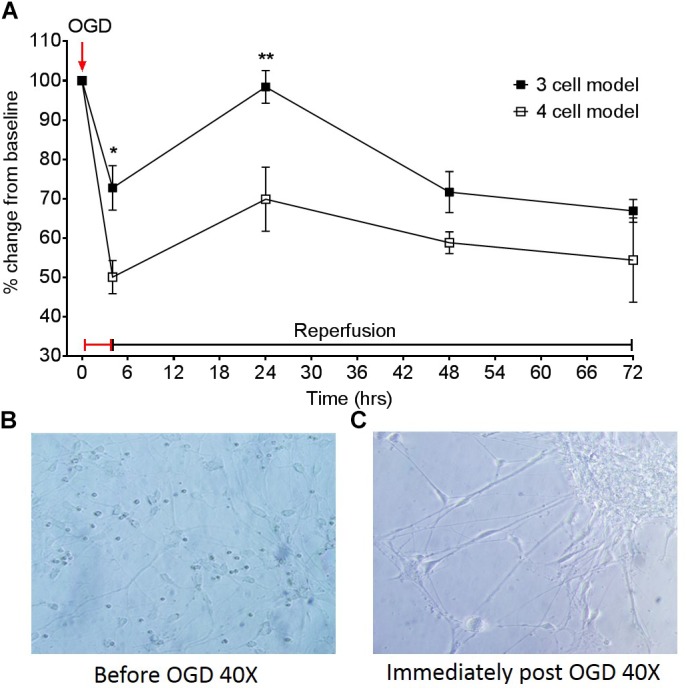
**(A)** Effect of a 4 h oxygen-glucose deprivation (OGD) protocol on transepithelial resistance (TEER) as a marker of barrier tightness in a three cell model (HBMECs, astrocytes, and pericytes) and a four cell model (HBMECs, astrocytes, pericytes, and neurons). Neuronal images taken from the four cell model **(B)** before OGD 40× and **(C)** neuronal images immediately post OGD 40×. Data given as mean ± SEM, *n* = 3–6 from two experimental repeats, calculated as a % change from baseline TEER readings. Statistical analysis was conducted using 2-way ANOVA with Sidak’s multiple comparisons test, ^∗^*P* < 0.05 and ^∗∗^*P* < 0.01 was considered significant.

To assess the effect of having different cells present, changes in TEER from models D and E shown in Figure 1, were compared following an OGD protocol. Figure 5A highlights the different responses of a three cell and four cell model in response to a 4 h OGD protocol, followed by a reperfusion period. The three-cell mode exhibited approximately a 30% drop in TEER from baseline after 4 h OGD. This contrasts to the four-cell model which exhibited a 50% drop in TEER post OGD and was significantly different to the three-cell model *P* < 0.05. After OGD, when reperfusion was initiated, TEER was able to return to baseline in the three-cell model, however, BBB permeability only marginally recovered by 20% in the four-cell model. This was significantly different at 24 h (*P* < 0.01) but not 48 or 72 h. Images 5B and C show light microscope images of neurons in the four-cell model before and immediately after the OGD protocol, respectively. In Figure 5C neuronal clumping is clearly visible along with apparent neurite fragmentation compared to Figure 5B showing healthy neurons prior to OGD.

### Dexamethasone Application

**FIGURE 6:**
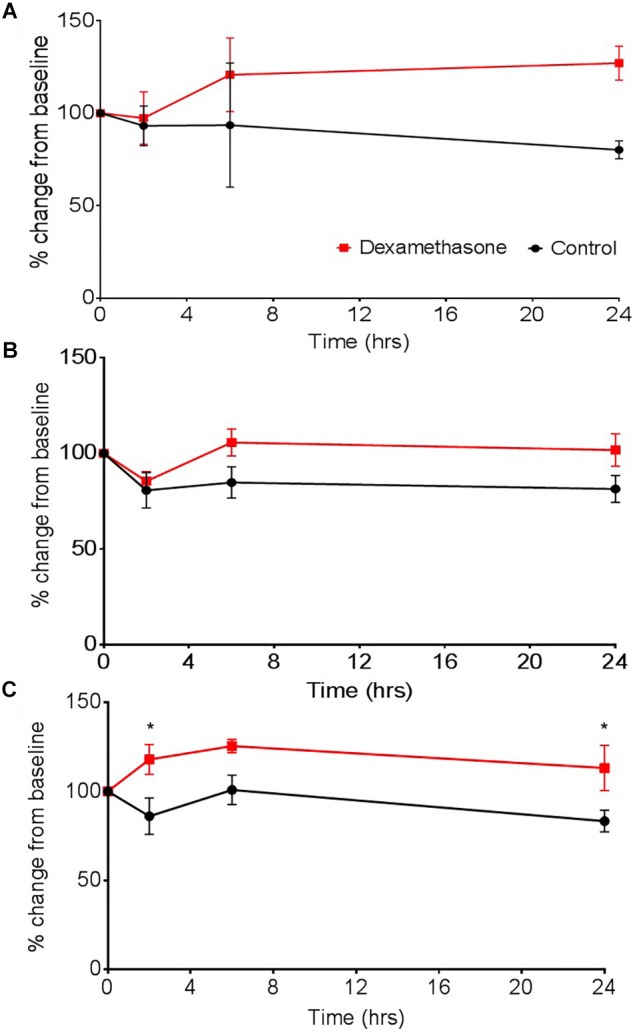
The effect of dexamethasone on transepithelial resistance (TEER) as a measure of barrier tightness in **(A)** a two cell (astrocytes and HBMECs), **(B)** three cell (astrocytes, HBMECs, and pericytes), and **(C)** four cell model (astrocytes, HBMECs, pericytes, and neurons). Dexamethasone (1 μM) was added to the luminal side and used as positive control that is known to decrease permeability, thus increase TEER. Data represented as mean ± SEM, *n* = 4 from two experimental repeats. Statistical analysis was conducted using 2-way ANOVA with Sidak’s multiple comparisons test, ^∗^*P* < 0.05 was considered significant.

Dexamethasone increased barrier tightness in all three models, as shown by increases in TEER and exhibiting overall significance as a result of drug interaction in the three cell and four cell model, *P* < 0.05. The two-cell model was the most unstable out of the three models, as shown by greater fluctuations and variability in TEER measurements (Figure 6A). The three-cell model was considerably more stable but differences in TEER between dexamethasone treated and control were only observed after 2 h (Figure 6B). The four-cell model was the fastest to exhibit an increase in barrier tightness (i.e., increased TEER) as a result of dexamethasone application (Figure 6C) and this reached significance compared to the vehicle control at 2 and 24 h (*P* < 0.05).

## Discussion

The BBB can be compromised in a range of different conditions, including but not limited to ischaemic stroke, Alzheimer’s disease, cancer, and multiple sclerosis (MS). Research into these disorders that affect the BBB is plagued by translational difficulty, resulting in many potential compounds and/or therapies failing to surpass phase I/II clinical trials. This is at least partly due to a lack of suitable *in vitro* models that can predict drug effectiveness pre-clinically. Most, if not all, current BBB models exhibit “pitfalls” whether that be cost, time or resources. Models that offer the closest representation of the BBB are often complex and expensive to replicate, adding to the cost of the drug screening process. To help improve the translatability of *in vitro* data, we developed a transwell style model that incorporates four primary human cell types, representing the NVU more than other BBB models currently available. We found that our novel four-cell model was superior in modeling ischaemic stroke and drug application *in vitro* compared to a three-cell and a two-cell model as shown through changes in TEER as a measure of barrier integrity and dexamethasone application.

### Implications for Drug Testing

The effect of dexamethasone was assessed in three transwell models; a two cell, three cell and four cell model. Greater instability in barrier strength and a slower response was exhibited by the two-cell model after dexamethasone application. This could also suggest that models containing just two cell types, in this case astrocytes and HBMECs, would also react differently to other drug applications and are therefore are not sufficient to truly model drug interactions at the BBB. Whilst the three-cell model shared the same trend in increasing barrier strength, it exhibited more stable TEER values compared to the two-cell model and dexamethasone treated wells were overall significantly different to the vehicle control. Also, by introducing pericytes (generating a three cell model) there was a large increase in baseline TEER from 30 to 40 Ω, again highlighting the role of pericytes in strengthening vascular stability at the BBB and the need for their presence in BBB models (Bergers and Song, 2005; Dohgu et al., 2005; Nakagawa et al., 2007; Ferland-McCollough et al., 2017) Interestingly, the four-cell model exhibited a significant increase in barrier tightness (as shown by an increase in TEER) compared to the vehicle control at just 2 h after dexamethasone application. Although neurons in this model do not directly interact with the BBB, neurons have been shown to secrete a number of vasoactive substances, including VEGF, which influence barrier forming properties and early angiogenesis (Engelhardt, 2003; Eichmann and Thomas, 2013). These comparison data highlight the variations in data obtained from models containing different cell types and the impact this can have on drug screening. This stresses the importance of having a more representative BBB model containing additional cells present at the NVU.

### Implications for Protocol Testing

Currently there are a wide range of *in vitro* BBB models available, but despite promising developments in modeling the BBB, there are still gaps in model design, primarily the inability to include all cell types present in the NVU. Whilst most transwell systems incorporate astrocytes and HBMECs, only more recent studies have introduced pericytes or neurons into these model designs. To gain a better understanding of how these cells contribute to the breakdown of the BBB in ischaemic conditions, we subjected our three cell and four cell models to an OGD protocol and measured TEER overtime to assess changes in barrier integrity. Interestingly, we found that with the presence of neurons our model exhibited a larger decrease in TEER compared to the three-cell model, which only contained astrocytes, pericytes and HBMECs. Similarly, whilst the three-cell model was able to recover 24 h post OGD the four-cell model only marginally recovered by approximately 20%, highlighting the role of and sensitivity of neurons in the level of damage ensued by the OGD protocol. Altogether, we have shown that with the addition of neurons our model became more vulnerable to damage; exhibiting a greater loss of barrier strength shown by a decrease in TEER, supporting previous work which showed that ischaemic neurons disrupt the endothelial barrier through increasing VEGF secretion (Li et al., 2014). Thus, omitting neurons from a BBB modeling stroke would underestimate the damage caused and contribution of neurons to the breakdown of the BBB post ischaemia.

### Limitations and Future Development

Although our model now includes four cell present in the NVU, our model does not incorporate flow which is an important feature to maintain the BBB phenotype *in vitro.* Studies have shown that sheer stress is critical to increase cell longevity and influence cell phenotype, regulate BBB transport, preventing de-differentiation (Desai et al., 2002; Chiu et al., 2005; Partyka et al., 2017). Culturing HBMECs under physiological shear stress, is particularly important in a ischaemic stroke setting because there is an interruption in blood flow. Microfluidic systems that mimic physiological flow have the advantage in that they can simulate continuous flow improving translation to the environment (Partyka et al., 2017; Wang et al., 2017).

Equally, there is increasing evidence of the role of microglial cells in BBB breakdown. These resident brain immune cells have been shown to release pro-inflammatory mediators that increase barrier permeability and reduce levels of certain TJs, thus playing a key role in BBB breakdown in pathological states (da Fonseca et al., 2014; Shigemoto-Mogami et al., 2018) Therefore, future work should assess whether additional cells and shear stress can be incorporated into transwell style models.

## Conclusion

The overall function of the NVU is the perfusion of brain tissue to supply neurons with essential nutrients and the ability of neurons to regulate this blood flow. Glia, namely astrocytes, act as mediators between the vascular and neural compartments (Lo et al., 2015). Pericytes provide an extra level of communication between the endothelia and astrocytes as well as serving a prominent immune function (Darland et al., 2003; Armulik et al., 2011; Kovac et al., 2011). During cerebral ischaemia, these complex interactions are disrupted, and homeostasis is lost as a consequence of functional, morphological and metabolic changes within the NVU (Lo et al., 2015). It is important to model how these cells interact in both normoxic and ischaemic conditions to study the pathophysiology of ischaemic stroke. Finally, transwell systems offer noticeable advantages over the more complex models as they maintain the ease simpler cell culture set up and often use minimal resources. We believe our model offers a closer representation of the BBB, whilst maintaining the ease of a transwell setup.

## Data Availability

The datasets generated for this study are available on request to the corresponding author.

## Author Contributions

NS performed the research. TE and SO’S designed the research study. NS and SO’S analyzed the data. NS, TE, and SO’S wrote the manuscript.

## Conflict of Interest Statement

The authors declare that the research was conducted in the absence of any commercial or financial relationships that could be construed as a potential conflict of interest.
